# Supplementary material for: Evaluation of Screening Program and Phylogenetic Analysis of SARS-CoV-2 Infections among Hospital Healthcare Workers in Liège, Belgium
Source: Viruses. 2022 Jun 14;14(6):1302. doi: 10.3390/v14061302 (PMC9227503; doi:10.3390/v14061302)
Supplement: Supplementary file 1 [file viruses-14-01302-s001.zip › viruses-1708054-supplementary.pdf]

Table S1. Number of SARS-CoV-2 reverse transcriptase-polymerase chain reaction (RT-PCR) tests performed per subject.

Number of SARS-CoV-2 RT-PCR Tests.	Number of Subject (%)
1	21 (3.0)
2	46 (6.5)
3	106 (15.1)
4	106 (15.1)
5	70 (10.0)
6	63 (9.0)
7	63 (9.0)
8	34 (4.8)
9	35 (4.9)
10	22 (3.1)
11	17 (2.4)
12	17 (2.4)
13	16 (2.3)
14	11 (1.6)
15	11 (1.6)
16	8 (1.1)
17	9 (1.3)
18	5 (0.7)
19	5 (0.7)
20	4 (0.6)
21	4 (0.6)
22	6 (0.9)
23	8 (1.1)
24	6 (0.9)
25	7 (1.0)
26	1 (0.1)
27	1 (0.1)
28	1 (0.1)

Table S2. Impact of demographic characteristics, healthcare workers (HCWs) staff role, and time since beginning of the study on reverse transcriptase-polymerase chain reaction (RT-PCR) result. Univariate and multivariate Generalized Estimating Equations (GEE) models.

**Characteristics**	**Univariate GEE Models**		**Final GEE Multivariate Model**	
	Coef. ± SE	*p*-value	Coef. ± SE	*p*-value
Age (years)	-0.0068 ± 0.0086	0.43	-	-
Gender (reference = Female)	0.025 ± 0.23	0.91	-	-
Height (cm)	0.0070 ± 0.0093	0.46	-	-
Weight (kg)	0.0052 ± 0.0065	0.43	-	-
BMI (kg/m^2)	0.012 ± 0.021	0.57	-	-
Smokers	-0.40 ± 0.32	0.21	-	-
Diabetes mellitus	-0.54 ± 0.49	0.19	-	-
Hypertension	-0.15 ± 0.38	0.69	-	-
Heart failure/coronary artery disease	-0.55 ± 1.0	0.60	-	-
Asthma	0.079 ± 0.29	0.78	-	-
Autoimmune disease	-0.75 ± 0.40	0.064	-	-
Immunodeficiency	-1.3 ± 0.82	0.11	-	-
Non hematological cancer	0.69 ± 0.59	0.24	-	-
Staff role<br>Control group: Administrative staff and research scientists	-	-	-	-
Laboratory staff handling respiratory samples	0.71 ± 0.32	0.028	1.3 ± 0.40	0.0018
Laboratory staff not handling respiratory samples	0.18 ± 0.33	0.58	0.18 ± 0.38	0.63
Physicians	0.29 ± 0.37	0.43	0.18 ± 0.38	0.65
Paramedics	1.1 ± 0.29	0.0002	0.82 ± 0.29	0.0042
Nurses	0.72 ± 0.26	0.0047	0.60 ± 0.29	0.036
Technicians	0.45 ± 0.44	0.31	0.49 ± 0.46	0.29

In contact with patients	0.42 ± 0.19	0.022	0.40 ± 0.29	0.18
Number of weeks since January 1, 2020	0.067 ± 0.0089	<0.0001	0.070 ± 0.0082	<0.0001

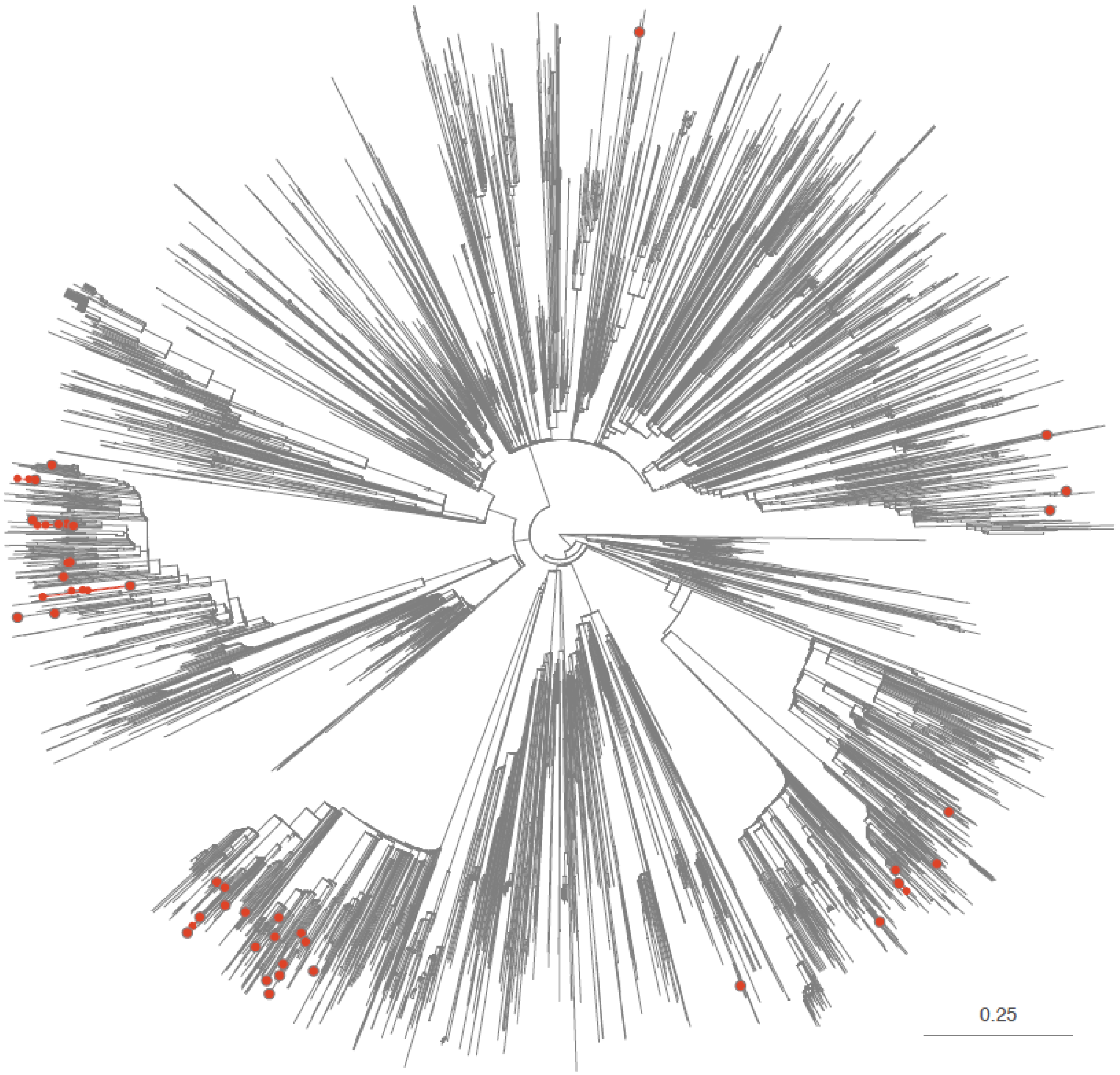

Figure S1. Time-scaled phylogeny in which we identified phylogenetic clades introduced in the University Hospital of Liège (Belgium) and delineated through a discrete phylogeographic reconstruction along the tree (while only considering two potential ancestral locations: "hospital" and "other location"). We identified a minimum of 35 introduction events into the hospital (95% highest posterior density interval = [36-38]) for 45 sequences sampled among healthcare workers (HCWs) from the hospital. On the phylogeny, large red nodes correspond to the most ancestral node of each clade resulting from an introduction event into the hospital. Most of these clades consist of only one sampled sequence: 30 (95% highest posterior density interval = [27-32]) out of 45 sequenced positive cases corresponded to independent introduction events into the hospital. In the figure, small red nodes correspond to sampled sequences that would not result from a distinct introduction event into the hospital. In other words, smaller red nodes are tip nodes belonging to clades gathering at least two sequences sampled among HCWs from the hospital. In the figure, smaller red nodes are tip nodes corresponding to sequences sampled among HCWs but that do not result from a distinct introduction event into the hospital. In other words, smaller red nodes correspond to clades gathering at least two sequences sampled among HCWs, and the phylogenetic branches of these clades are also highlighted in red.
